# Supplementary material for: Menarche, pubertal timing and the brain: female-specific patterns of brain maturation beyond age-related development
Source: Biol Sex Differ. 2024 Mar 26;15:25. doi: 10.1186/s13293-024-00604-4 (PMC10964568; doi:10.1186/s13293-024-00604-4)
Supplement: Supplementary file 1 — Additional file 1. Methods; Tables S1, S2; Figures S1, S2, S3, S4. [file 13293_2024_604_MOESM1_ESM.docx]

**Menarche, pubertal timing and the brain: female-specific patterns of brain maturation beyond age-related development**

Nina Gottschewsky^1,#,*^, Dominik Kraft^1,#^, Tobias Kaufmann^1,2,3*^

*^1^ Department of Psychiatry and Psychotherapy, Tübingen Center for Mental Health, University of Tübingen, Germany*

*^2^ Centre for Precision Psychiatry, Division of Mental Health and Addiction, Institute of Clinical Medicine, University of Oslo, Oslo, Norway*

*^3^ German Center for Mental Health (DZPG), Partner site Tübingen, Germany*

*# These authors contributed equally*

* Correspondence: Nina Gottschewsky, MSc and Tobias Kaufmann, PhD

Email:

[nina.gottschewsky@maxplanckschools.de](mailto:nina.gottschewsky@maxplanckschools.de)

[tobias.kaufmann@med.uni-tuebingen.de](mailto:tobias.kaufmann@med.uni-tuebingen.de)

Postal address:

Department of Psychiatry and Psychotherapy, Calwerstr. 14, 72076 Tübingen

**Additional File 1**

**Methods**

Description of in- and exclusion procedure

We initially chose data from the second year follow up visit since it provided the highest and best-balanced number of pre- vs postmenarcheal (n_pre_= 2860, n_post_= 1848) female participants. After data exclusion due to missing MRI data, puberty data, and relevant demographic data, as well as due to inadequate imaging quality, we had data from n= 2075 pre- vs. n=1173 post-menarcheal female participants available. We then randomly sampled 80% of the data into the training set, resulting in n_pre_= 1656 and n_post_=942 in the training set. To balance the group sizes (i.e., pre- vs. post-menarche) as well as the age and scan site distribution in the training set, we performed a propensity score matching, which resulted in a final and balanced training set of n_pre_ = 775 and n_post_= 775 female participants. The remaining 20% of subjects were allocated to the holdout test sample, resulting in n_pre_=419 vs n_post_= 231 subjects for holdout performance assessment.

**Tables**

*Table S1: Cortical and subcortical features for menarche classification and brain age prediction.*

| Thickness | Volume | Area | SubcorticalVolumes |
| --- | --- | --- | --- |
| bankssts_thicknessstd | bankssts_volume | bankssts_area | smri_vol_scs_ltventriclelh |
| caudalanteriorcingulate_thicknessstd | caudalanteriorcingulate_volume | caudalanteriorcingulate_area | smri_vol_scs_inflatventlh |
| caudalmiddlefrontal_thicknessstd | caudalmiddlefrontal_volume | caudalmiddlefrontal_area | smri_vol_scs_crbwmatterlh |
| cuneus_thicknessstd | cuneus_volume | cuneus_area | smri_vol_scs_crbcortexlh |
| entorhinal_thicknessstd | entorhinal_volume | entorhinal_area | smri_vol_scs_tplh |
| fusiform_thicknessstd | fusiform_volume | fusiform_area | smri_vol_scs_caudatelh |
| inferiorparietal_thicknessstd | inferiorparietal_volume | inferiorparietal_area | smri_vol_scs_putamenlh |
| inferiortemporal_thicknessstd | inferiortemporal_volume | inferiortemporal_area | smri_vol_scs_pallidumlh |
| isthmuscingulate_thicknessstd | isthmuscingulate_volume | isthmuscingulate_area | smri_vol_scs_3rdventricle |
| lateraloccipital_thicknessstd | lateraloccipital_volume | lateraloccipital_area | smri_vol_scs_4thventricle |
| lateralorbitofrontal_thicknessstd | lateralorbitofrontal_volume | lateralorbitofrontal_area | smri_vol_scs_bstem |
| lingual_thicknessstd | lingual_volume | lingual_area | smri_vol_scs_hpuslh |
| medialorbitofrontal_thicknessstd | medialorbitofrontal_volume | medialorbitofrontal_area | smri_vol_scs_amygdalalh |
| middletemporal_thicknessstd | middletemporal_volume | middletemporal_area | smri_vol_scs_aal |
| parahippocampal_thicknessstd | parahippocampal_volume | parahippocampal_area | smri_vol_scs_ltventriclerh |
| paracentral_thicknessstd | paracentral_volume | paracentral_area | smri_vol_scs_inflatventrh |
| parsopercularis_thicknessstd | parsopercularis_volume | parsopercularis_area | smri_vol_scs_crbwmatterrh |
| parsorbitalis_thicknessstd | parsorbitalis_volume | parsorbitalis_area | smri_vol_scs_crbcortexrh |
| parstriangularis_thicknessstd | parstriangularis_volume | parstriangularis_area | smri_vol_scs_tprh |
| pericalcarine_thicknessstd | pericalcarine_volume | pericalcarine_area | smri_vol_scs_caudaterh |
| postcentral_thicknessstd | postcentral_volume | postcentral_area | smri_vol_scs_putamenrh |
| posteriorcingulate_thicknessstd | posteriorcingulate_volume | posteriorcingulate_area | smri_vol_scs_pallidumrh |
| precentral_thicknessstd | precentral_volume | precentral_area | smri_vol_scs_hpusrh |
| precuneus_thicknessstd | precuneus_volume | precuneus_area | smri_vol_scs_amygdalarh |
| rostralanteriorcingulate_thicknessstd | rostralanteriorcingulate_volume | rostralanteriorcingulate_area | smri_vol_scs_aar |
| rostralmiddlefrontal_thicknessstd | rostralmiddlefrontal_volume | rostralmiddlefrontal_area | smri_vol_scs_ccps |
| superiorfrontal_thicknessstd | superiorfrontal_volume | superiorfrontal_area | smri_vol_scs_ccmidps |
| superiorparietal_thicknessstd | superiorparietal_volume | superiorparietal_area | smri_vol_scs_ccct |
| superiortemporal_thicknessstd | superiortemporal_volume | superiortemporal_area | smri_vol_scs_ccmidat |
| supramarginal_thicknessstd | supramarginal_volume | supramarginal_area | smri_vol_scs_ccat |
| frontalpole_thicknessstd | frontalpole_volume | frontalpole_area |  |
| temporalpole_thicknessstd | temporalpole_volume | temporalpole_area |  |
| transversetemporal_thicknessstd | transversetemporal_volume | transversetemporal_area |  |
| insula_thicknessstd | insula_volume | insula_area |  |

Note: Naming refers to labels obtained in Freesurfer 7 in the PNC cohort. For the ABCD study, we used the same features, yet some may have been labelled differently in the ABCD data base.

*Table S2: Results of association analyses when including BMI, SES and race / ethnicity as covariates.*

|  | **variable** | **coef** | **p-value** |
| --- | --- | --- | --- |
|  |  |  |  |
| Menarche class probabilities residualised for age and scanner ~ BAG residualised for age and scanner + BMI + SES + race / ethnicity + constant | | | |
|  | BAG_resid_ | 0.0093 | <0.001 |
|  | BMI | 0.0051 | 0.034 |
|  | SES | -0.0293 | 0.061 |
|  | race/ethnicity _asian_ | 0.1535 | 0.010 |
|  | race/ethnicity _black_ | 0.0720 | 0.038 |
|  | race/ethnicity _hispanic_ | -0.0445 | 0.092 |
|  | race/ethnicity _white_ | -0.1345 | <0.001 |
|  | race/ethnicity _other_ | -0.0744 | 0.028 |
|  | constant | -0.0278 | 0.525 |
| Menarche class probabilities ~ age at menarche + age + scanner + BMI + SES + race / ethnicity + constant | | | |
|  | age at menarche | -0.0533 | 0.083 |
|  | age | 0.0157 | < 0.001 |
|  | BMI | -0.0015 | 0.767 |
|  | SES | -0.0138 | 0.655 |
|  | race/ethnicity _asian_ | -0.2429 | 0.097 |
|  | race/ethnicity _black_ | -0.0271 | 0.191 |
|  | race/ethnicity _hispanic_ | -0.1192 | 0.421 |
|  | race/ethnicity _white_ | -0.2617 | 0.898 |
|  | race/ethnicity _other_ | -0.2478 | 0.975 |
|  | constant | -0.8986 | 0.028 |
|  |  | | |
| Menarche class probabilities ~ Pubertal Status_(c/y)_+ age + scanner + BMI + SES + race / ethnicity + constant | | | |
| Caregiver-reported | Pubertal Status | 0.0261 | 0.008 |
|  | age | 0.0071 | <0.001 |
|  | BMI | 0.0025 | 0.368 |
|  | SES | -0.0333 | 0.054 |
|  | race/ethnicity _asian_ | 0.0844 | 0.302 |
|  | race/ethnicity _black_ | -0.0238 | 0.656 |
|  | race/ethnicity _hispanic_ | -0.0945 | 0.047 |
|  | race/ethnicity _white_ | -0.2745 | <0.001 |
|  | race/ethnicity _other_ | -0.1850 | <0.001 |
|  | constant | -0.4934 | 0.010 |
| Youth-reported | Pubertal Status | 0.0106 | 0.250 |
|  | BMI | 0.0054 | 0.044 |
|  | SES | -0.0311 | 0.068 |
|  | age | 0.0075 | <0.001 |
|  | race/ethnicity _asian_ | 0.0661 | 0.405 |
|  | race/ethnicity _black_ | -0.0170 | 0.752 |
|  | race/ethnicity _hispanic_ | -0.1034 | 0.028 |
|  | race/ethnicity _white_ | -0.2842 | <0.001 |
|  | race/ethnicity _other_ | -0.1034 | <0.001 |
|  | constant | -0.5245 | 0.006 |

Note: BMI = Body Mass Index, SES = Socioeconomic Status, Pubertal Status_(c/y)_ = pubertal status category reported by either caregiver or youth. MRI scanner dummy variables were omitted from the table for legibility but were included in the modeling.

**Figures**


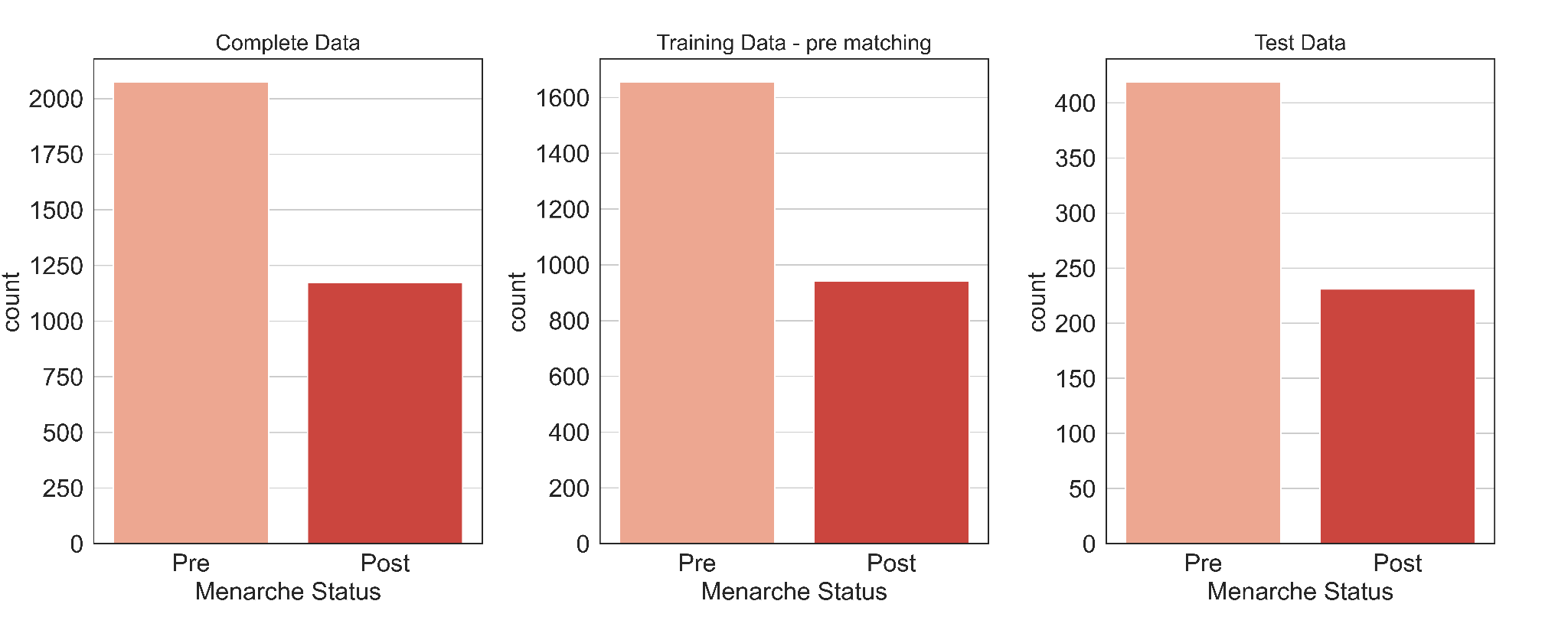


Figure S1: Distribution of menarche status prior to propensity score matching.


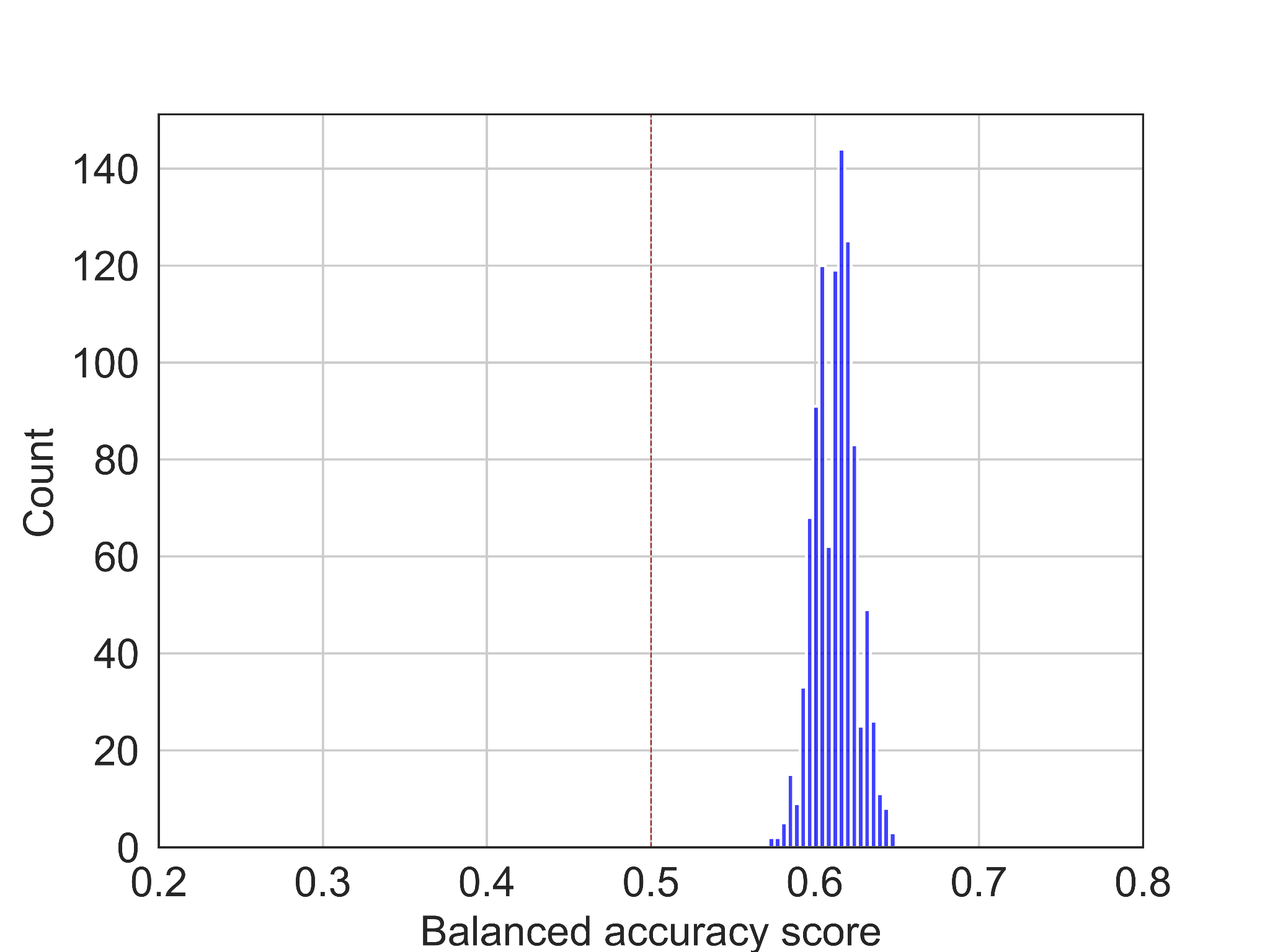


Figure S2: Balanced accuracy score distribution across 1000 random samplings of pre-menarcheal girls to derive a balanced test set. Red line indicates the 50% chance level.


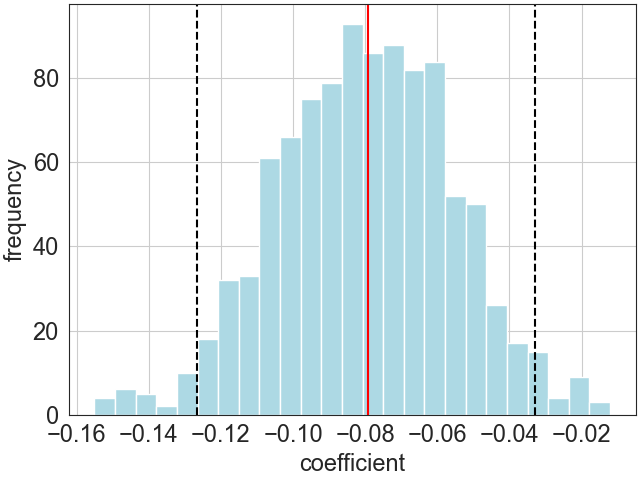


Figure S3: Distribution of coefficients across 1000 bootstrapped OLS regression models of the form Menarche_class_probabilities ~ Age_at_menarche + age + scanner + constant. Red line shows the coefficient value of the original model. Black dotted lines show the 95% confidence interval (boostrapped 95% CI [-0.127, -0.033]).


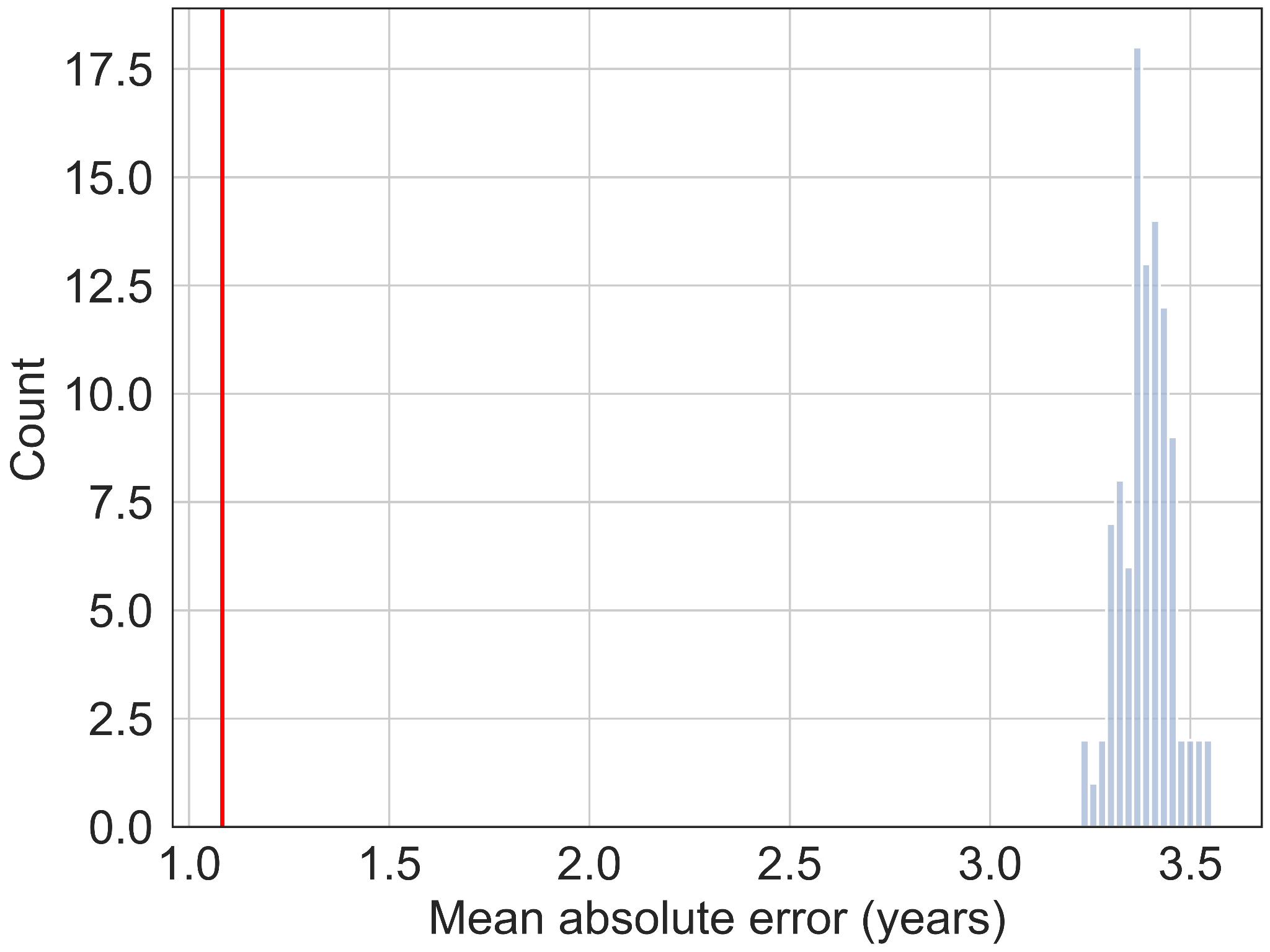


Figure S4: Mean absolute error (MAE) distribution across 1000 random permutations of the brain age model. Model was trained on 1000 permutations of training data with shuffled labels and used to predict test data. The red line indicates the empirical MAE.
